# Prognostic and Clinicopathological Value of Human Leukocyte Antigen G in Gastrointestinal Cancers: A Meta-Analysis

**DOI:** 10.3389/fonc.2021.642902

**Published:** 2021-05-12

**Authors:** Yongjia Peng, Jian Xiao, Wenyun Li, Shuna Li, Binbin Xie, Jiang He, Chaoqun Liu

**Affiliations:** ^1^ Department of Nutrition, School of Medicine, Jinan University, Guangzhou, China; ^2^ Department of Medical Oncology, The Sixth Affiliated Hospital, Sun Yat-sen University, Guangzhou, China; ^3^ Department of Statistics, School of Medicine, Jinan University, Guangzhou, China; ^4^ Department of Mathematics and Physics, School of Biomedical Engineering, Southern Medical University, Guangzhou, China

**Keywords:** prognosis, meta-analysis, HLA-G antigens, gastrointestinal, cancer

## Abstract

**Background:**

The prognostic value of human leukocyte antigen G (HLA-G) expression in gastrointestinal (GI) cancers remains controversial. Thus, this meta-analysis aimed to summarize available evidence from case-control or cohort studies that evaluated this association.

**Methods:**

The PubMed, EMBASE, Cochrane Library, and Web of Science databases were searched to identify relevant studies written in English published up to April 1, 2021, and with no initial date. Furthermore, the Google Scholar and Google databases were also searched manually for gray literature. The protocol for this meta-analysis was registered at PROSPERO (CRD42020213411). Pooled hazard ratios (HRs) or odds ratios (ORs) and 95% confidence intervals (CIs) were estimated for end points using fixed- and random-effects statistical models to account for heterogeneity. Publication bias was evaluated using a funnel plot, Begg’s and Egger’s tests, and the “trim and fill” method.

**Results:**

A total of 30 eligible articles with 5737 unique patients, including 12 studies on colorectal cancer (CRC), 6 on gastric cancer (GC), 5 on esophageal cancer (ESCC), 5 on hepatocellular carcinoma (HCC), and 2 on pancreatic adenocarcinoma (PC), were retrieved. Both univariate (HR = 2.01, 95% CI: 1.48 ~ 2.72) and multivariate (HR = 2.69, 95% CI: 2.03 ~ 3.55) analyses revealed that HLA-G expression was significantly correlated with poor overall survival (OS), regardless of the cancer type or antibody used. Subgroup analysis stratified by antibody showed that the 4H84 (*I^2^* = 45.8%, *P* = 0.101) antibodies could be trustworthy and reliable for detecting HLA-G expression in GI cancers. In addition, HLA-G expression was found to be correlated with adverse clinicopathological parameters such as clinical stage, nodal status, metastasis, and histological grade but not tumor status.

**Conclusion:**

Elevated HLA-G expression indicates a poor prognosis for GI cancer patients, and screening for this marker could allow for the early diagnosis and treatment of GI cancers to improve survival rates.

## Introduction

Gastrointestinal (GI) cancers, composed of esophageal, colorectal, pancreatic, stomach, and liver cancers, are the most common malignancies worldwide, accounting for one-quarter of the global cancer incidence. In 2018, the estimated number of GI cancer-related deaths reached 3.4 million, accounting for one-third (35%) of the total deaths (1). Recent studies have predicted that the number of GI cancer-related deaths will increase by 73% to 5.6 million by 2040 (2, 3). Advances in cancer treatment have markedly improved the clinical outcomes of colorectal cancer (CRC) and gastric cancer (GC), including relief of symptoms and prolonged survival (4, 5), and have therefore decreased their mortality rates. However, some of these tumors and high-risk adenomas are potentially curable if they are detected and removed at an early stage. The five-year survival rate of CRC ranges from 91% for patients with localized disease down to 71% and 14% for patients with regional and distant metastasis, respectively (6). Therefore, novel biomarkers to improve cancer diagnosis and prognosis are crucial for reducing cancer burden and mortality.

Human leukocyte antigen G (HLA-G), a nonclassical HLA class I molecule, includes at least 4 membrane-bound subtypes (HLA-G1-HLA-G4) and 3 soluble subtypes (HLA-G5-HLA-G7). Recent evidence has shown that HLA-G has direct immunosuppressive effects on natural killer (NK) cells, dendritic cells (DCs), and T cells and that HLA-G can induce tolerogenic regulatory CD4(+)CD25(+)FoxP3(+) T cells, DCs, and NK cells, which provide these immune effectors with long-term immunomodulatory functions (7). In various malignancies, abnormal HLA-G expression in cancer lesions or increased levels of circulating HLA-G (sHLA-G) have long been observed (8–19). Generally, HLA-G expression in tumor tissue is detected by immunohistochemistry (IHC) or Western blotting (WB) using antibodies such as 4H84, 5A6G7, HGY, MEM-G/1 or MEM-G/2, and plasma sHLA-G levels are quantified using a commercial ELISA kit. HLA-G expression is well established as a mechanism used by tumor cells to escape host immune surveillance and maintain their survival and growth; for example, HLA-G expression can allow tumor cells to escape cytotoxic T lymphocyte-mediated recognition and destruction (20). The potential relationship between tumor cells and their microenvironment, including immune system components, plays an important role in the development, growth and spread of GI cancers (21–23). Studies on the prognostic value of HLA-G and its association with clinicopathological parameters in GI cancer patients have presented conflicting results (24–39). Some studies have demonstrated that positive HLA-G expression or increased levels of circulating sHLA-G are associated with unfavorable survival (24, 25, 27–29, 32, 34, 35, 37, 39), while others have found no significant association (31, 36, 38, 40, 41). Some studies (12, 24, 28, 41, 42) have reported that HLA-G expression in GI cancer patients is correlated with certain clinicopathological characteristics, while others (32, 35, 36, 38, 43) have reached the opposite conclusion. However, it remains uncertain whether HLA-G can be used as a marker for GI cancer, so comprehensive analysis and related studies are still needed. In addition, the limited predictive power of the traditional staging system was due to its reliance only on tumor cell characteristics but ignored the host immune response against cancers (44). Therefore, patient stratification based on both traditional staging and molecular profiling of prognostic biomarkers is warranted to improve the diagnosis and prognosis of cancer patients and to refine the treatment protocol. We expect that HLA-G can be used as a convenient, accurate and low-cost test for adjuvant diagnosis of patients, as well as for large-scale population screening for GI cancer.

Therefore, we conducted a systematic review to combine the best available evidence from identified individual studies, with the intention of elucidating the prognostic value of HLA-G and the association of HLA-G with the clinicopathological parameters of GI cancer patients under various settings, to arrive at some more certain conclusions.

## Material and Methods

### Data Sources and Search Strategy

This meta-analysis was performed in accordance with the Preferred Reporting Items for Systematic Review and Meta-Analyses (PRISMA) statement. The protocol was registered in the PROSPERO database (CRD42020213411), and the PRISMA checklist is attached as **Supplementary Table 1**.

A comprehensive search of the literature from the PubMed, EMBASE, Cochrane Library, and Web of Science databases was conducted for studies written in English published up to April 1, 2021, and with no initial date. Google Scholar and the Google databases were also searched manually for gray literature. The terms used in this search were colorectal cancer/colon and rectal cancer (CRC), colon cancer (COAD), rectal cancer (RC), stomach cancer/gastric cancer (GC), esophageal cancer (ESCC), pancreatic cancer/pancreatic adenocarcinoma (PC), liver cancer/hepatocellular carcinoma (HCC), small bowel cancer (SBC), gastrointestinal cancer (GI), and human leukocyte antigen G (HLA-G), combined using Boolean operators “AND” and “OR”. Two investigators (YJP and WYL) independently screened the titles, abstracts, and full texts of all articles for eligibility and determined the articles for final inclusion by group consensus. The detailed literature search strategy is presented in **Supplementary Table 2**.

### Inclusion and Exclusion Criteria

The original studies identified from the search results were screened for eligibility according to the following inclusion criteria: (1) patients in the studies should have a confirmed diagnosis of GI cancer; (2) the studies should include HLA-G expression in tumor tissue or soluble HLA-G in plasma or serum measured before treatment; and (3) the studies should include the associations between the expression of HLA-G in the tissue or serum and the overall survival (OS), progression-free survival (PFS), metastasis-free survival (MFS) or clinicopathological parameters. The exclusion criteria were as follows: (1) duplicates; (2) narrative reviews, meta-analyses or conference abstracts; (3) cell research or animal-based research; and (4) studies without insufficient data.

### Data Extraction

Two authors (YP and WL) extracted data independently in duplicate from all eligible studies using a prespecified standard data extraction form including the following information: first author’s name, year of publication, country, sample size, tumor type, sex, age, HLA-G measurement technique, measure of HLA-G or sHLA-G (positive/negative, strong/weak, or high/low), depth of invasion (tumor status), nodal status, metastasis status, tumor-node-metastasis (TNM) stage, histological differentiation (tumor grade), follow-up time, and hazard ratios (HRs) with 95% confidence intervals (CIs) for both univariate and multivariate analyses. GI cancer was diagnosed by pathological examination in each study. For accuracy, all data were cross-checked against the original publications. In case of missing data, attempts were made to contact the corresponding authors. Studies were excluded if no replies were received.

### Quality Assessment

The quality of each study was evaluated using the guidelines of the Newcastle-Ottawa Scale (NOS) by two reviewers independently. Each study was assessed based on three major aspects, namely, selection, comparability, and exposure, with a score ranging from 0 to 9. Studies awarded scores of six or higher were considered high quality. Disagreements between the review authors over the risk of bias were resolved by a third author and discussed until a consensus was reached.

### Outcome Measures

The primary outcome measures were the expression level in tumor tissues and plasma levels of HLA-G. HLA-G expression was calculated according to the staining intensity of positive cells using various antibodies. HLA-G was also measured directly in serum or plasma. The secondary outcome measures were clinicopathological parameters, such as clinical stage, tumor status (T), nodal status (N), metastasis (M), and histological grade.

### Statistical Methods

Stata (version 11.0; Stata Corporation, College Station, TX, USA) was used for all statistical tests. HRs and 95% CIs were extracted from all included articles to estimate the prognostic value of HLA-G in GI cancers. Sensitivity analysis was performed to assess the stability of the meta-analysis. A cumulative meta-analysis was conducted to identify any trend in the estimates over time. The *I^2^* statistic and the Cochrane *Q* test were used to quantify statistical heterogeneity when P < 0.050 for the χ^2^ test, and *I^2^* > 50% indicated statistical heterogeneity between studies. When warranted, the random-effects model was applied for pooling. Sensitivity analysis was also performed to identify which article was the main determinant of the pooled result and the main source of heterogeneity. Subsequent subgroup analyses were performed to explore the between-study heterogeneity. Publication bias was evaluated by funnel plots, and Egger’s and Begg’s tests were used to assess funnel plot asymmetry. Trim and fill techniques were considered n cases of substantial publication bias.

## Results

### Characteristics of the Included Studies

**Figure 1:**
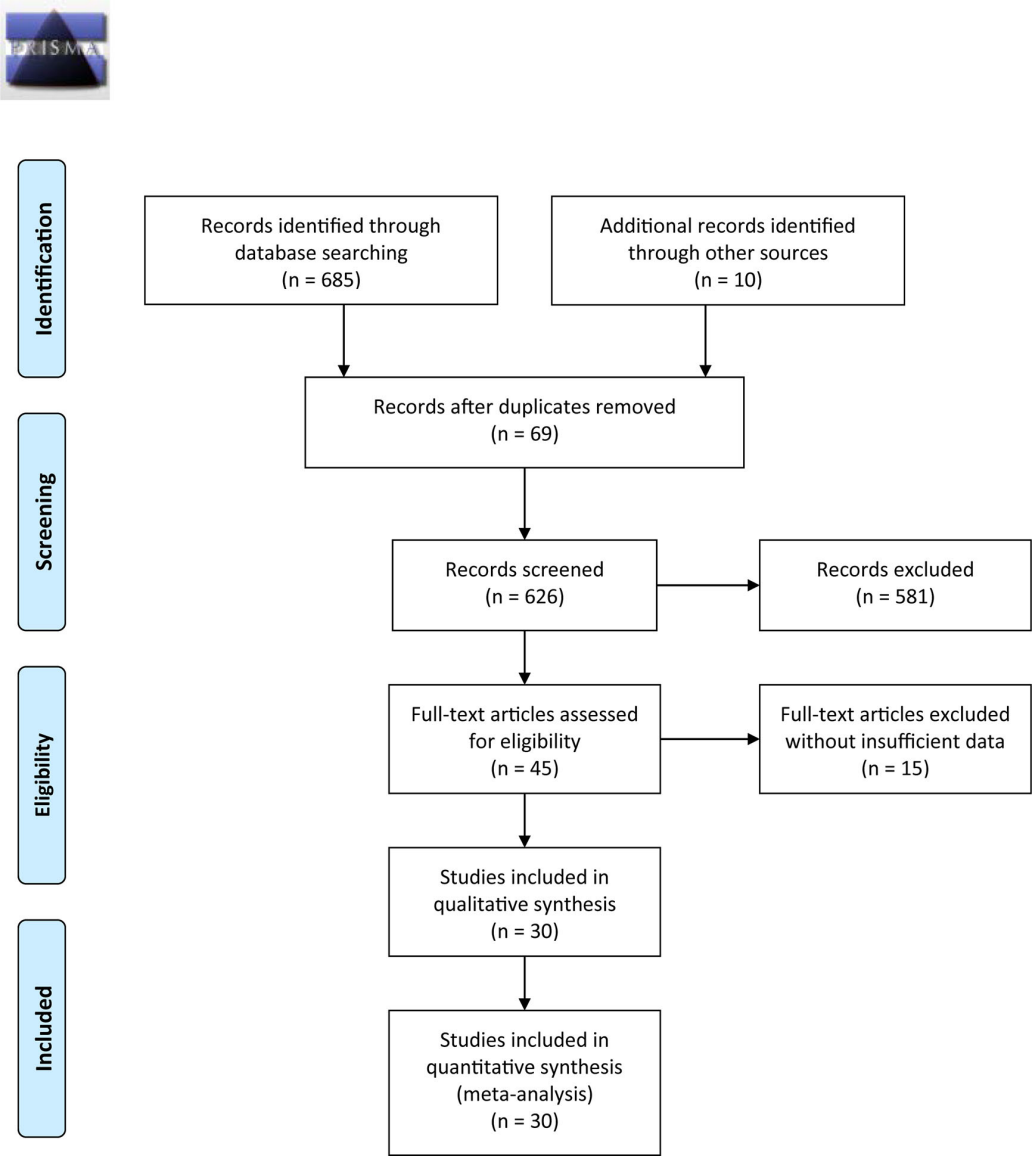
Flow chart of the literature search and selection of studies.

**Figure 1** outlines the detailed process of study selection. Following the prespecified search strategy, a total of 695 publications were obtained from all the databases, with 626 studies remaining after removing duplicates. After reading the titles and/or abstracts, 581 were further excluded for various reasons, and 30 eligible studies were ultimately included in this systematic review. These articles were published between 1998 and 2020, among which 20 were conducted in China, 3 were conducted in Japan, 1 was conducted in Turkey, 2 were conducted in the Netherlands, 2 were conducted in Iran, 1 was conducted in Australia, and 1 was conducted in Italy. Of the 30 articles included in the meta-analysis, there were 12 studies on CRC (24, 30–36, 38, 43, 45, 46), 6 studies on GC (25, 28, 39, 41, 42, 47), 5 studies on ESCC (7, 12, 48–50), 5 studies on HCC (26, 27, 29, 37, 51), and 2 studies on PC (52, 53). Among them, one study specifically addressed COAD (31), one study addressed RC (30), and one addressed both HCC and PAAD (37). The total sample size reached 5737, including 3738 CRC, 649 GC, 441 ESCC, 611 HCC, and 298 PC samples.

Most studies investigated tumor tissue HLA-G expression (n = 24), two studies (34, 46) detected plasma sHLA-G levels, and others (33) measured both plasma and tumor tissue levels. The average score of the eligible studies on the NOS was 7.0. The basic characteristics of the included studies are summarized in **Table 1**.

**Table 1:** Characteristics of the included studies.

year	Study ID	Country	Cancer type	Sample size ^a^	Male %	Age (years)	Detection method	Antibody	Comparison	Follow up (months)	UnivariateHR (95% CI)	MultivariateHR (95% CI)	NOS
1998	Fukushima	Japan	CRC	39	NA	NA	IHC	HLA-G/GST	Pos/Neg	NA	NA	NA	6
2006	Ishigami	Japan	GC	115	68.7	Mean: 61, range: 31 ~ 82	IHC	MEM-G/1	Pos/Neg	60	NA	NA	7
2007	Yie	China	ESCC	121	81.8	Mean: 58, SD: 12	IHC	HGY	Pos/Neg	mean: 24, range: 4~36	3.33 (1.74, 6.37)	2.99 (1.52, 5.88)	7
2007	Ye	China	CRC	201 (85)	52.7	Mean: 64, SD: 13	IHC	HGY	Pos/Neg	Mean: 27, range: 3 ~ 36	4.60 (1.76, 12.00)	3.14 (1.34, 8.10)	7
2007	Yie	China	GC	160 (101)	71.9	Mean: 63, SD: 11	IHC	HGY	Pos/Neg	Mean: 26, range: 4~36	5.72 (2.50, 13.10)	9.08 (3.44, 24.00)	7
2009	Cai	China	HCC	173	85.0	Median 52	IHC	MEM-G/1	Low/High	NA	NA	1.99 (1.24, 3.18)	8
2010	Lin	China	HCC	219	84.9	Median 52	IHC/ ELISA	4H84	Pos/Neg	NA	NA	NA	6
2011	Wang	China	HCC	36	83.3	Mean:49, range: 30 ~ 67	WB/ ELISA	MEM-G/1	Pos/Neg	NA	4.57 (1.04, 19.98)	NA	7
2011	Lin	China	ESCC	79	70.9	Median 58	IHC	4H84	Pos/Neg	mean:12 range: 3 ~ 36	3.76 (1.73 ~ 8.16)	3.83 (1.73 ~ 8.44)	8
2011	Du	China	GC	179	73.2	≤60: 108, >60: 71	IHC	4H84	Pos/Neg	Mean: 21, range: 4 ~ 79	NA	NA	8
2013	Zeng	China	HCC	109	94.5	Median: 58, range: 17 ~ 72	IHC	NA	Pos/Neg	Median: 60, range: 3 ~ 17	NA	2.631 (1.590 ~ 4.353)	6
2013	Hu	China	ESCC	60	60.0	Median: 59, range: 34 ~ 76	IHC	NA	Pos/Neg	NA	NA	NA	7
2013	Tuncel	Turkey	GC	52	59.6	Median: 63, range: 33 ~ 87	IHC	5A6G7	Pos/Neg	Max: 75	3.12 (1.23, 6.22)	2.66 (1.24, 5.72)	6
2014	Zheng	China	ESCC	60	78.3	Mean: 60, SD: 11	IHC /ELISA	MEM-G/1	Pos/Neg	NA	NA	NA	6
2014	Zeestraten	Netherlands	Colon	285	48.1	<50: 32; ≥50: 251	IHC	4H84	Pos/Neg	Max: 220	1.20 (0.90, 1.80)	NA	6
2014	Reimers	Netherlands	Rectal	484	64.0	Mean: 64.5, SD: 11	IHC	4H84	Strong/Weak	Max:170	0.76 (0.58, 1.01)	0.88 (0.66, 1.19)	8
2015	Zhou	China	PC	158	86.08	Median: 62, range: 35 ~ 85	IHC	NA	Pos/Neg	NA	NA	NA	8
2015	Guo	China	CRC	102	58.8	<60: 49, ≥60: 53	IHC	MEM-G/2	Pos/Neg	Max: 60	2.17 (1.08, 4.35)	3.22 (1.35, 7.69)	6
2017	Samadi	Iran	CRC	100	59.0	Mean: 51, SD: 15	IHC	4H84	Pos/Neg	Max: 250	1.55 (1.05, 2.30)	1.62 (1.02, 2.57)	6
2017	Zhang	China	CRC	457 (417)	58.6	Median: 66, range: 26 ~ 90	IHC	4H84	Pos/Neg	Median: 47, range: 1 ~ 103	1.35 (0.93, 1.95)	1.42 (0.98, 2.06)	7
2017	Zhang^2^								Strong/Weak (55% cut-off)		1.43 (1.01, 2.02)	1.48 (1.04, 2.10)	
2017	Kirana	Australia	CRC	133	57.1	<65: 61; ≥65: 72	ELISA	NA	High/Mod /Low	Max: 80	NA	NA	8
2017	Kirana^2^	Australia	CRC	255	55.9	< 65: 116; ≥ 65: 138	IHC	4H84	Strong/Weak	Max: 250	NA	0.57 (0.20, 1.65)	
2017	Li	China	CRC	178	56.2	range: 28 ~ 86	ELISA	NA	High/Low	Median: 47, range: 2 ~ 91	1.87 (1.21, 2.90)	1.62 (1.01, 2.62)	8
2017	Wan	China	GC	49	67.4	< 60: 17, ≥ 60: 32	IHC	4H84	Pos/Neg	NA	NA	NA	7
2018	Shahraki	Iran	HCC	74	75.7	Mean: 45, SD: 11	IHC	4H84	Pos/Neg	NA	NA	NA	4
2018	Shahraki	Iran	PC	42	47.6	Mean: 57, SD: 12	IHC	4H84	Pos/Neg	NA	NA	NA	
2018	Xu	China	ESCC	121	81.8	Median: 58	IHC	HGY	Pos/Neg	mean: 26, range: 4 ~ 36	NA	2.99 (1.52 ~ 5.88)	5
2018	Lin	China	CRC	379 (341)	56.5	Median: 66	IHC	4H84	Pos/Neg	Median: 45	1.27 (0.83, 1.93)	NA	8
2018	Lin^2^							5A6G7	Pos/Neg	Median: 45	0.81 (0.56, 1.18)	NA	
2018	Murdaca	Italy	GC	94	59.6	Mean: 72, SD: 10	IHC	4H84	Pos/Neg	Mean: 61, range: 13 ~ 97	NA	4.41 (2.48, 7.86)	8
2019	Cai	China	CRC	88	77.3	Mean: 67, range:20 ~ 90	IHC	4H84	Pos/Neg	Median: 60	NA	NA	8
2020	Hiraoka	Japan	PC	98	63.3	<65: 52, ≥65: 46	IHC	4H84	Pos/Neg	Median: 66, range: 3 ~ 201	2.03 (1.22, 3.36)	1.85 (1.10, 3.12)	8
2020	Jiao	China	CRC	1037	58.1	Mean: 62, SD: 13	ELISA	NA	High/Low	Max: 60	1.91 (1.38, 2.31)	1.78 (1.32, 2.28)	9

a. Numbers in brackets are the numbers of patients followed up in the prognosis evaluation.

b. The 2017 Zhang study used 2 different percentages of stained cells as cutoffs: 5% to classify a sample as positive/negative and 55% to classify a sample as strong/weak.

c. The 2017 Kirana study included 133 plasma samples and 255 archival CRC tumor tissue samples and measured plasma sHLA-G by ELISA and HLA-G expression by IHC.

d. The 2018 Shahraki study included patients with both hepatocellular carcinoma (HCC) and pancreatic adenocarcinoma (PAAD) tumors.

e. The 2018 Lin study compared two antibodies for HLA-G detection in the same samples.

NA, not available; IHC, immunohistochemistry; WB, Western blotting; CRC, colorectal cancer; GC, gastric cancer; ESCC, esophageal cancer, ESCC, esophageal cancer; PAAD, pancreatic cancer; HCC, hepatocellular carcinoma; HRs, hazard ratios; CIs, confidence intervals; NOS, Newcastle-Ottawa Scale.

### Association Between HLA-G and the Prognosis of GI Cancers

Fourteen studies (6 on CRC, 3 on GC, 3 on ESCC, 1 on PC, and 2 on HCC) detected HLA-G by IHC, 12 of which conducted univariate analysis and multivariate analysis of OS and disease-free survival (DFS).

**Figure 2:**
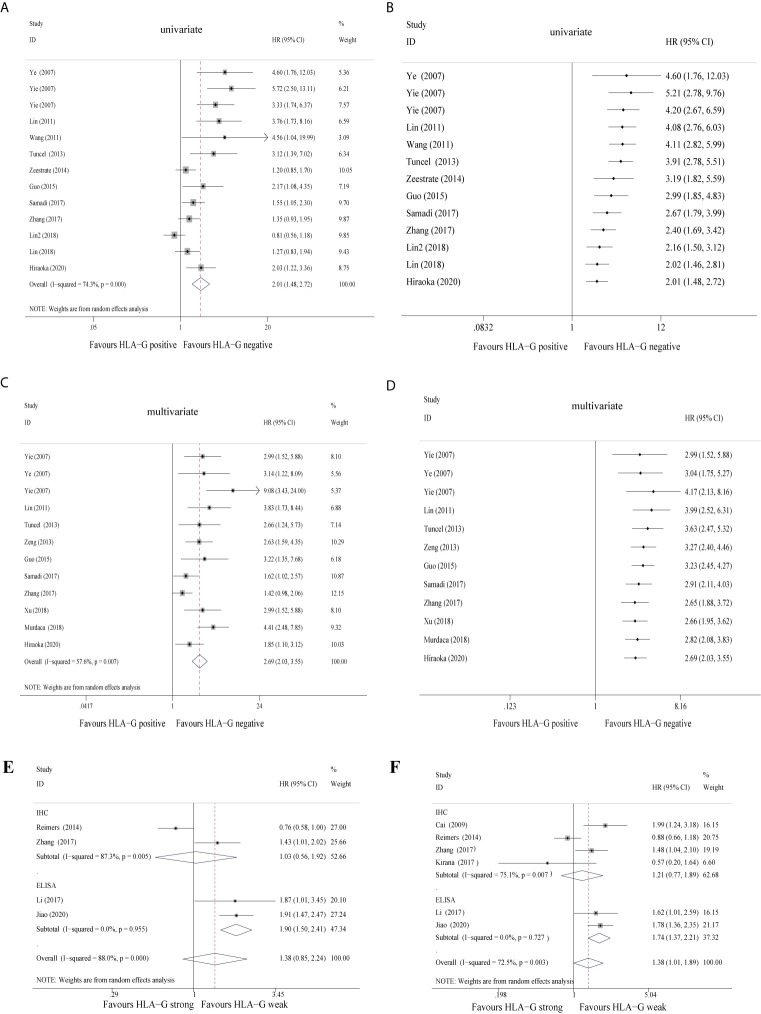
Forest plot of primary outcomes in the studies in the meta-analysis and cumulative meta-analysis.

When the 12 studies were pooled with the univariate random-effects model, the single-arm meta-analysis showed that the pooled HR was 2.01 (95% CI: 1.48 ~ 2.72) (**Figure 2A**), while the cumulative meta-analysis demonstrated no remarkable temporal effect (**Figure 2B**). Significant heterogeneity was found among the 12 included studies (*P* < 0.001, *I^2^* = 74.3%) (**Figure 2A**). Subgroup analysis stratified by cancer site showed that HLA-G-positive status predicted a poor prognosis in CRC (HR = 1.39, 95% CI: 1.05 ~ 1.83), GC (HR = 4.20, 95% CI: 2.32 ~ 7.60), ESCC (HR = 3.50, 95% CI: 2.13 ~ 5.76), and PC (HR = 2.03, 95% CI: 1.22 ~ 3.36) patients (**Supplementary Figure S1**). Subgroup analysis stratified by antibody showed that HLA-G positivity was associated with a poor prognosis, regardless of which antibody was used (HGY (HR = 4.20, 95% CI: 2.67 ~ 6.59), 4H84 (HR = 1.52, 95% CI: 1.20 ~ 1.96), 5A6G7 (HR = 1.51, 95% CI: 0.40 ~ 5.65), MEM-G/1 (HR = 4.56, 95% CI: 1.04 ~ 19.97), and MEM-G/2 (HR = 2.17, 95% CI: 1.08 ~ 4.36)). The heterogeneity of HLA-G expression detected by the 4H84 (*I^2^* = 45.8%, *P* = 0.101) and HGY (*I^2^* = 0%, *P* = 0.589) antibodies was quite low, whereas it was high with the 5A6G7 antibody (*I^2^* = 88.6%, *P* = 0.003) (**Supplementary Figure S1**).

Furthermore, when the 12 studies were pooled with the multivariate random-effects model, the single-arm meta-analysis showed that the pooled HR was 2.69 (95% CI: 2.03 ~ 3.55) (**Figure 2C**), while the cumulative meta-analysis also identified no time dependence of the outcomes (**Figure 2D**). A moderate level of heterogeneity was found across the 12 included studies (*P* = 0.007, *I^2^* = 57.6%) (**Figure 2C**). Subgroup analysis stratified by cancer type revealed that HLA-G-positive patients exhibited a worse prognosis than HLA-G-negative patients under the fixed-effects model (the pooled HRs were 1.70 (95% CI: 1.31 ~ 2.21) in CRC, 4.33 (95% CI: 2.86 ~ 6.57) in GC and 3.19 (95% CI: 2.12 ~ 4.81) in ESCC). The heterogeneity of each cancer type was not statistically significant (CRC, *P* = 0.201, *I^2^* = 35.2%; GC, *P* = 0.150, *I^2^* = 47.2%; ESCC, *P* = 0.871, *I^2^* = 0.0%) (**Supplementary Figure S1**). Despite adjustments for potential confounding factors, the pooled HRs did not change significantly (**Figure 2C**). In the pooled multivariate analysis, HLA-G expression was also associated with a poor prognosis in GI cancer patients, regardless of which antibody was used (**Supplementary Figure S1**). These results again indicated that compared to negative HLA-G expression, positive HLA-G expression was related to shorter overall survival in GI cancer patients. Another five studies (30, 33, 36, 51) divided the HLA-G expression level into two groups (high or low) according to the IHC staining intensity (strong or weak). In the univariate and multivariate analyses, the pooled HRs for HLA-G expression were 1.38 (95% CI: 0.85 ~ 2.24) (**Figure 2E**) and 1.38 (95% CI: 1.01~ 1.89) (**Figure 2F**), respectively. In brief, high/strong HLA-G expression was associated with poor overall survival in patients with GI cancer. Due to the lack of literature with this grouping method, the overall heterogeneity was high.

### Association Between HLA-G Expression and the Clinicopathological Parameters of GI Cancer Patients

**Figure 3:**
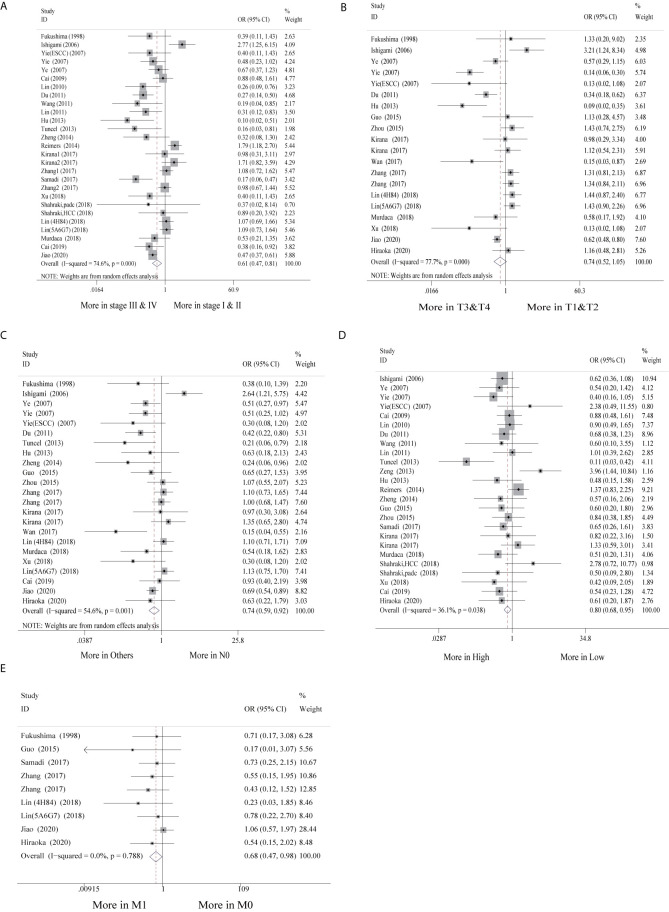
Forest plot of other outcomes in the studies in the meta-analysis.

Of the 30 studies, 28 studies (28/30, 93.3%) with clinicopathological parameters were included for further meta-analysis. The clinicopathological information on GI cancer patients is shown with pooled odds ratios (ORs) and 95% CIs in **Table 2**. According to the results, HLA-G expression had statistically significant associations with most clinicopathological parameters, such as clinical stage (I-II vs. III-IV: OR = 0.61, 95% CI: 0.47 ~ 0.81, *P* < 0.001, *I^2^* = 74.6%), nodal status (N_0_ vs. N_1-2_: OR = 0.74, 95% CI: 0.59 ~ 0.92, *P* < 0.001, *I^2^* = 54.6%), metastasis (M_0_ vs. M_1_: OR = 0.68, 95% CI: 0.47 ~ 0.98, *P* = 0.788, *I^2^* = 0.0%), and histological grade (high vs. low: OR = 0.80, 95% CI: 0.68 ~ 0.95, *P* = 0.038, *I^2^* = 36.1%), but was not associated with tumor status (T_1-2_ vs. T_3-4_: OR = 0.74, 95% CI: 0.52 ~ 1.05, *P* < 0.001, *I^2^* = 77.7%) (**Figure 3**).

**Table 2:** Meta‐analysis of the relationship between HLA-G expression and clinicopathological parameters.

Clinicopathological parameters	Studies (n)	Sample size	OR	LCI	UCI	Heterogeneity		
						I^2^	*P*_h_	model
Clinical stage	22	4538	0.61	0.47	0.81	74.6%	<0.001	random
T (tumor status)	17	3758	0.74	0.52	1.05	77.7%	<0.001	random
N (nodal status)	20	3958	0.74	0.59	0.92	54.6%	0.001	random
M (metastasis)	7	2212	0.68	0.47	0.98	0.00%	0.788	fixed
histological grade	25	4337	0.80	0.68	0.95	36.1%	0.038	fixed

OR, odds ratio; LCI, lower limits of the confidence interval; UCI, upper limits of the confidence interval.

Additionally, subgroup analyses stratified by cancer type showed that high HLA-G expression was associated with end-stage disease (OR = 0.30, 95% CI: 0.17 ~ 0.53, *P* = 0.716, *I^2^* = 0%), advanced tumor status (OR = 0.11, 95% CI: 0.04 ~ 0.30, *P* = 0.926, *I^2^* = 0%), and positive nodal status (OR = 0.36, 95% CI: 0.18 ~ 0.69, *P* = 0.747, *I^2^* = 0%) in ESCC patients, but no significant association was observed in other cancers (**Supplementary Figure S2**). For the HLA-G antibody subgroup analysis, HLA-G expression detected by the HGY antibody was significantly correlated with clinical stage, tumor status, and nodal status (*P* < 0.050), while HLA-G expression detected by the 4H84 antibody was only related to clinical stage and metastasis (*P* < 0.050) (**Supplementary Figure 2**).

### Publication Bias and Sensitivity Analyses

As shown in **Figure S3**, the funnel plots were slightly asymmetric, and Begg’s and Egger’s tests and the “trim and fill” method further confirmed the existence of a marginal publication bias in the included articles (**Supplementary Table 3** and **Supplementary Figure 4**). Sensitivity analyses were performed to ensure the robustness of the results, and they did not show any significant change in these pooled effects (**Supplementary Figure 5**).

## Discussion

This meta-analysis of 30 eligible studies (including 5737 GI cancer cases) supported a positive association between HLA-G expression and the risk of GI cancers. Although subgroup analysis revealed that different cancer types and HLA-G-detecting antibodies served as the main sources of heterogeneity, the results also demonstrated that high HLA-G expression was associated with a poor prognosis in GI cancer patients, regardless of which antibody was used for detection. Furthermore, the overexpression of HLA-G was highly correlated with several clinicopathological parameters (clinical stage, nodal status, metastasis, and histological grade) in GI cancers.

In 1998, Fukushima (43) first reported the ectopic expression and significant upregulation of HLA-G gene expression in GI cancer compared with normal controls, and the immunomodulatory function of HLA-G has since been extensively studied. A series of studies evaluated the association between HLA-G expression and the prognosis of GI cancers. However, their conclusions were paradoxical. Ye et al. (24) carried out a prospective cohort study and found that patients with HLA-G-positive tumors had significantly shorter survival times than patients with HLA-G-negative tumors. Similar results were found in other types of GI cancers, including ESCC (7, 12, 49), GC (25, 28, 39), and HCC (27, 51), among others. However, a few studies (36, 40, 41) have demonstrated that no significant correlation exists between HLA-G expression and the prognosis of GI cancers. However, Reimers et al. (30) reported the opposite result; their findings indicated that weak HLA-G expression was related to poor OS and DFS in RC patients. Interestingly, Lin et al. demonstrated that HLA-G expression was related to a poor prognosis when detected with antibody 4H84, but the results were inconsistent when using the 5A6G7 antibody. The latest meta-analysis from 2019 (54) reported a significant relationship between the HLA-G 14-bp ins/del polymorphism and a decreased overall cancer risk. Compared with this meta-analysis, our study mainly focused on GI cancers and utilized a more powerful and detailed analysis approach to reveal a significant association between HLA-G expression and an increased risk of GI cancers. Currently, although the molecular mechanisms underlying GI cancer development and progression are not yet fully clarified, a growing body of evidence indicates that GI cancers can be partly mediated by both oxidative stress (OS) and microRNA (miRNA) involvement (55). Additionally, several miRNAs have been reported to modulate HLA-G expression by targeting its 3′UTR target, including miR-148a, miR-148b and miR-152 (56). Thus, the HLA-G expression level can be used as a reliable prognostic marker for GI cancers.

Because the invasive nature of the disease and the tumor microenvironment are different across multiple GI diseases (36, 57–60), the expression of HLA-G varies among different cancer types. Thus, we further conducted a stratified analysis to examine the relationship between HLA-G expression and GI cancers by cancer type. The subgroup analysis results were consistent with the combined analysis of GI cancers, showing that the expression of HLA-G was associated with a poor prognosis in CRC, GC, and ESCC. In the CRC subgroup, the reliability of the results was quite high due to the large number of studies included and low heterogeneity. We acknowledge that the number of studies was relatively small in other subgroups; however, the heterogeneity of GC and ESCC was extremely low with a sufficient sample size. This might have certain guiding significance for clinical practice, but more accurate research in different GI cancer types is still needed. In the multivariate analysis, the combined HR remained unchanged, which supported the conclusion that HLA-G expression was associated with a poor prognosis in GI cancer patients. HLA-G can help tumor cells evade the immune system by inhibiting NK cells and T cell activation and is overexpressed in many malignancies. However, different cancer types express HLA-G at different levels or in different forms. For example, in the analysis of HLA-G expression in different GI cancer types, the HLA-G protein expression rate differed among cancers; specifically, the expression rate, as determined by IHC, was 25 – 70% in CRC (32, 35, 36), 25 – 74% in GC (25, 28, 39), and 66 – 91% in ESCC (7, 12, 50). The current meta-analysis, which included a larger sample size, provides a more precise evaluation of the association between positive HLA-G expression and the poor prognosis of GI cancers.

Due to the use of different HLA-G antibodies (39), the prognostic value of HLA-G is still controversial, so it is worth considering how the antibodies used contribute to the heterogeneity. Lin’s study revealed that the expression of HLA-G detected by the 4H84 antibody was different from that detected by the 5A6G7 antibody, and the antibody analyses even yielded the opposite conclusions. A previous study (38) also reported conflicting results; specifically, the expression of HLA-G was detected by the 5A6G7 antibody, but HLA-G was not detected by the 4H84 antibody in some renal cell carcinoma (RCC) cases. Hence, we further conducted a stratified analysis to examine the heterogeneity due to the different antibodies used. Interestingly, the expression of HLA-G detected by the 4H84 and HGY antibodies was closely related to a poor prognosis in GI cancer patients, while that detected by the 5A6G7 antibody was not related to the prognosis of GI cancer patients. The reason for the discrepancy may be that the diverse antibodies used in these studies may recognize different epitopes on the HLA-G protein. For example, the 4H84 antibody recognizes an epitope in the HLA-G α1 domain, the MEM-G/1 antibody reacts with the denatured HLA-G heavy chain, the MEM-G/2 antibody recognizes all free heavy chains of HLA-G subtypes, and the 5A6G7 antibody recognizes an epitope encoded by intron 4. In addition, the 4H84 antibody has also been confirmed by international conferences as a reference tool for evaluating HLA-G expression in paraffin-embedded specimens (7). In the analysis, the expression of HLA-G detected by the 4H84 antibody was associated with a poor prognosis in GI cancers, so 4H84 may be a reliable marker of HLA-G expression in GI cancers. Due to the limited number of studies, conclusions regarding other antibodies could not be justifiable.

Twenty-eight included studies with clinical data were also extracted to explore the relationship between HLA-G expression and GI cancer progression, and the results were mixed. Researchers (25) found that HLA-G expression was significantly correlated with clinicopathological features, such as clinical stage, location, histological grade, depth of invasion, and lymph node metastasis. However, Farjadian et al. (58) reported the opposite result, namely, that HLA-G expression was not related to any clinicopathological factors. Furthermore, other studies (26, 35, 36, 41, 49) showed that HLA-G expression was only associated with certain clinicopathological characteristics. However, no comprehensive evaluation of the association of HLA-G expression and the clinicopathological features of GI cancer patients has been published to date. This meta-analysis showed that there was a significant correlation between HLA-G expression and TNM stage, lymph node status, tumor depth, and lymph node metastasis but not the depth of tumor invasion. Interestingly, subgroup analysis showed that HLA-G expression was only associated with clinicopathological features in ESCC, while no significant correlation was observed in other types of tumors. Similarly, several studies showed that HLA-G expression was significantly higher in ESCC tissues than in normal esophageal epithelial cells (12, 50). Therefore, HLA-G has the potential to serve as a biomarker for ESCC prognosis.

This meta-analysis has several limitations. First, the methods of quantifying HLA-G positivity varied widely in the included studies. For example, in some reports, HLA-G expression was calculated according to the staining intensity of positive cells, while others calculated expression according to the percentage of positive cells, or even a combination of the two parameters. Second, although the total sample size of this meta-analysis was relatively large, the sample sizes of the stratified analysis were relatively small, which might weaken the statistical power of the results. Third, all the studies included were observational studies, so substantial heterogeneity was inevitable in this meta-analysis due to the various regimens, doses, durations, center settings, populations and sample sizes. Therefore, sensitivity analyses were conducted to confirm the stability of the results. Ultimately, the funnel plots and Egger’s and Begg’s tests suggested a slight publication bias in the current study; since only published studies written in English were searched, other eligible studies may have been inadvertently excluded. The limited number of studies regarding the different cancer types or detected antibodies included in the subgroup analysis was insufficient to justify definitive conclusions. Considering the above limitations, the findings of this meta-analysis should be interpreted with caution. Therefore, further large-scale studies on different populations and different cancer types are required to validate the findings.

## Conclusion

In conclusion, the results of the current meta-analysis suggest that HLA-G expression is very likely associated with the clinical features and prognosis of GI cancers. Currently, the 4H84 antibody is a widely used and reliable detection method for HLA-G expression in GI cancers. Further studies, either large, prospective, randomized, controlled trials or basic molecular biological studies, are warranted to validate these findings in the future.

## Data Availability Statement

The original contributions presented in the study are included in the article/**Supplementary Material**. Further inquiries can be directed to the corresponding authors.

## Author Contributions

Study concept and design: CL, JX, and WL. Data extraction and analysis: YP, WL, SL, and BX. Manuscript drafting: YP, JH, and CL. All authors contributed to the article and approved the submitted version.

## Funding

This work was supported by the National Natural Science Foundation of China (No. 81903294) and the Natural Science Foundation of Guangdong Province (No. 2018030310412).

## Conflict of Interest

The authors declare that the research was conducted in the absence of any commercial or financial relationships that could be construed as a potential conflict of interest.

## Supplementary Material

Supplementary Figure 1
**(A, C)** Subgroup analysis of the correlation between HLA-G expression and overall survival (OS) in patients with gastrointestinal (GI) cancer according to the different cancer types. **(B, D)** Subgroup analysis of the correlation of HLA-G expression with OS in patients with GI cancer according to the different antibodies used for detection.

Supplementary Figure 2
**(A, C, E)** Subgroup analysis of the correlation between HLA-G expression and clinical stage, tumor status or nodal status for different cancer types. **(B, D, F)** Subgroup analysis of the correlation between HLA-G expression and clinical stage, tumor status or nodal status for different cancer types.

Supplementary Figure 3 Funnel plot: **(A)** positive vs. negative HLA-G expression (univariate analysis), **(B)** positive vs. negative HLA-G expression (multivariate analysis), **(C)** strong vs. weak HLA-G expression (univariate analysis), **(D)** strong vs. weak HLA-G expression (multivariate analysis), **(E)** clinical stage, **(F)** tumor status, **(G)** nodal status, **(H)** metastasis, **(I)** histological grade.

Supplementary Figure 4 Sensitivity of all outcomes. **(A)** positive vs. negative HLA-G expression (univariate analysis), **(B)** positive vs. negative HLA-G expression (multivariate analysis), **(C)** strong vs. weak HLA-G expression (univariate analysis), **(D)** strong vs. weak HLA-G expression (multivariate analysis), **(E)** clinical stage, **(F)** tumor status, **(G)** nodal status, **(H)** metastasis, **(I)** histological grade.

Supplementary Figure 5 Trim and fill of all outcomes. **(A)** positive vs. negative HLA-G expression (univariate analysis), **(B)** positive vs. negative HLA-G expression (multivariate analysis), **(C)** clinical stage, **(D)** tumor status, **(E)** nodal status, **(F)** metastasis, **(G)** histological grade.

## References

[1] Arnold M Abnet CC Neale RE Vignat J Giovannucci EL McGlynn KA. Global Burden of 5 Major Types of Gastrointestinal Cancer. Gastroenterology (2020) 159(1):335–49.e15. 10.1053/j.gastro.2020.02.068 32247694 PMC8630546

[2] Wang Y Li Z Xu S Guo J. Novel Potential Tumor Biomarkers: Circular RNAs and Exosomal Circular RNAs in Gastrointestinal Malignancies. J Clin Lab Anal (2020) 34:(7):e23359. 10.1002/jcla.23359 32419229 PMC7370736

[3] Ferlay J Colombet M Soerjomataram I Mathers C Parkin DM. Estimating the Global Cancer Incidence and Mortality in 2018: GLOBOCAN Sources and Methods. Int J Cancer (2019) 144: (8):1941–53. 10.1002/ijc.31937 30350310

[4] Ciombor KK Wu C Goldberg RM. Recent Therapeutic Advances in the Treatment of Colorectal Cancer. Annu Rev Med (2015) 66:83–95. 10.1146/annurev-med-051513-102539 25341011

[5] Salem ME Hartley M Unger K Marshall JL. Neoadjuvant Combined-Modality Therapy for Locally Advanced Rectal Cancer and Its Future Direction. Oncology (Williston Park) (2016) 30(6):546–62. 27306709

[6] American Cancer Society. Colorectal Cancer Facts & Figures 2017–2019. (2017) Atlanta: American Cancer Society. Available at: https://wwwcancerorg/content/dam/cancer-org/research/cancer-facts-and-statistics/colorectal-cancer-facts-and-figures/colorectal-cancer-facts-and-figures-2017-2019pdf.

[7] Lin A Zhang X Zhou WJ Ruan YY Xu DP Wang Q. Human Leukocyte Antigen-G Expression is Associated With a Poor Prognosis in Patients With Esophageal Squamous Cell Carcinoma. Int J Cancer (2011) 129(6):1382–90. 10.1002/ijc.25807 21128238

[8] Paul P Rouas-Freiss N Khalil-Daher I Moreau P Riteau B Le Gal FA. HLA-G Expression in Melanoma: A Way for Tumor Cells to Escape From Immunosurveillance. Proc Natl Acad Sci USA (1998) 95(8):4510–5. 10.1073/pnas.95.8.4510 PMC22520 9539768

[9] Bukur J Malenica B Huber C Seliger B. Altered Expression of Nonclassical HLA Class Ib Antigens in Human Renal Cell Carcinoma and its Association With Impaired Immune Response. Hum Immunol (2003) 64(11):1081–92. 10.1016/j.humimm.2003.08.350 14602239

[10] Davidson B Elstrand MB McMaster MT Berner A Kurman RJ Risberg B. HLA-G Expression in Effusions is a Possible Marker of Tumor Susceptibility to Chemotherapy in Ovarian Carcinoma. Gynecol Oncol (2005) 96(1):42–7. 10.1016/j.ygyno.2004.09.049 15589578

[11] Yie SM Hu Z. Human Leukocyte Antigen-G (HLA-G) as a Marker for Diagnosis, Prognosis and Tumor Immune Escape in Human Malignancies. Histol Histopathol (2011) 26(3):409–20. 10.14670/hh-26.409 21210353

[12] Yie SM Yang H Ye SR Li K Dong DD Lin XM. Expression of HLA-G is Associated With Prognosis in Esophageal Squamous Cell Carcinoma. Am J Clin Pathol (2007) 128(6):1002–9. 10.1309/jncw1qldfb6am9we 18024326

[13] Ugurel S Rebmann V Ferrone S Tilgen W Grosse-Wilde H Reinhold U. Soluble Human Leukocyte Antigen–G Serum Level is Elevated in Melanoma Patients and is Further Increased by Interferon-Alpha Immunotherapy. Cancer (2001) 92(2):369–76. 10.1002/1097-0142(20010715)92:2<369::aid-cncr1332>3.0.co;2-u 11466692

[14] Leleu X Le Friec G Facon T Amiot L Fauchet R Hennache B. Total Soluble HLA Class I and Soluble HLA-G in Multiple Myeloma and Monoclonal Gammopathy of Undetermined Significance. Clin Cancer Res (2005) 11(20):7297–303. 10.1158/1078-0432.ccr-05-0456 16243800

[15] Gros F Sebti Y de Guibert S Branger B Bernard M Fauchet R. Soluble HLA-G Molecules Increase During Acute Leukemia, Especially in Subtypes Affecting Monocytic and Lymphoid Lineages. Neoplasia (2006) 8(3):223–30. 10.1593/neo.05703 PMC1578523 16611416

[16] Sebti Y Le Maux A Gros F De Guibert S Pangault C Rouas-Freiss N. Expression of Functional Soluble Human Leucocyte Antigen-G Molecules in Lymphoproliferative Disorders. Br J Haematol (2007) 138(2):202–12. 10.1111/j.1365-2141.2007.06647.x 17593027

[17] Yie SM Yang H Ye SR Li K Dong DD Lin XM. Expression of Human Leucocyte Antigen G (HLA-G) is Associated With Prognosis in non-Small Cell Lung Cancer. Lung Cancer (2007) 58(2):267–74. 10.1016/j.lungcan.2007.06.011 17673327

[18] He X Dong DD Yie SM Yang H Cao M Ye SR. HLA-G Expression in Human Breast Cancer: Implications for Diagnosis and Prognosis, and Effect on Allocytotoxic Lymphocyte Response After Hormone Treatment In Vitro. Ann Surg Oncol (2010) 17(5):1459–69. 10.1245/s10434-009-0891-9 20052552

[19] Zhu CB Wang CX Zhang X Zhang J Li W. Serum sHLA-G Levels: A Useful Indicator in Distinguishing Colorectal Cancer From Benign Colorectal Diseases. Int J Cancer (2011) 128(3):617–22. 10.1002/ijc.25372 20473865

[20] Marincola FM Jaffee EM Hicklin DJ Ferrone S. Escape of Human Solid Tumors From T-Cell Recognition: Molecular Mechanisms and Functional Significance. Adv Immunol (2000) 74:181–273. 10.1016/s0065-2776(08)60911-6 10605607

[21] Fuchs A Colonna M. Innate Lymphoid Cells in Homeostasis, Infection, Chronic Inflammation and Tumors of the Gastrointestinal Tract. Curr Opin Gastroenterol (2013) 29(6):581–7. 10.1097/MOG.0b013e328365d339 24100718

[22] Moehler M Delic M Goepfert K Aust D Grabsch HI Halama N. Immunotherapy in Gastrointestinal Cancer: Recent Results, Current Studies and Future Perspectives. Eur J Cancer (2016) 59:160–70. 10.1016/j.ejca.2016.02.020 27039171

[23] Kobayashi H Enomoto A Woods SL Burt AD Takahashi M Worthley DL. Cancer-Associated Fibroblasts in Gastrointestinal Cancer. Nat Rev Gastroenterol Hepatol (2019) 16(5):282–95. 10.1038/s41575-019-0115-0 30778141

[24] Ye SR Yang H Li K Dong DD Lin XM Yie SM. Human Leukocyte Antigen G Expression: As a Significant Prognostic Indicator for Patients With Colorectal Cancer. Mod Pathol (2007) 20(3):375–83. 10.1038/modpathol.3800751 17277760

[25] Yie SM Yang H Ye SR Li K Dong DD Lin XM. Expression of Human Leukocyte Antigen G (HLA-G) Correlates With Poor Prognosis in Gastric Carcinoma. Ann Surg Oncol (2007) 14(10):2721–9. 10.1245/s10434-007-9464-y 17564748

[26] Lin A Chen HX Zhu CC Zhang X Xu HH Zhang JG. Aberrant Human Leucocyte Antigen-G Expression and its Clinical Relevance in Hepatocellular Carcinoma. J Cell Mol Med (2010) 14(8):2162–71. 10.1111/j.1582-4934.2009.00917.x PMC3823007 19799650

[27] Wang Y Ye Z Meng XQ Zheng SS. Expression of HLA-G in Patients With Hepatocellular Carcinoma. Hepatobiliary Pancreat Dis Int (2011) 10(2):158–63. 10.1016/s1499-3872(11)60025-8 21459722

[28] Tuncel T Karagoz B Haholu A Ozgun A Emirzeoglu L Bilgi O. Immunoregulatory Function of HLA-G in Gastric Cancer. Asian Pac J Cancer Prev (2013) 14(12):7681–4. 10.7314/apjcp.2013.14.12.7681 24460353

[29] Zeng XC Zhang T Chen W Chen GZ Li H Zhang Q. Human Leukocyte Antigen-G and Prognosis of Liver Transplantation in Patients With Hepatocellular Carcinoma. Chin J Tissue Eng Res (2013) 17(5):825–31. 10.3969/j.issn.2095-4344.2013.05.010

[30] Reimers MS Engels CC Putter H Morreau H Liefers GJ van de Velde CJ. Prognostic Value of HLA Class I, HLA-E, HLA-G and Tregs in Rectal Cancer: A Retrospective Cohort Study. BMC Cancer (2014) 14:486. 10.1186/1471-2407-14-486 24997850 PMC4094545

[31] Zeestraten EC Reimers MS Saadatmand S Goossens-Beumer IJ Dekker JW Liefers GJ. Combined Analysis of HLA Class I, HLA-E and HLA-G Predicts Prognosis in Colon Cancer Patients. Br J Cancer (2014) 110(2):459–68. 10.1038/bjc.2013.696 PMC3899753 24196788

[32] Guo ZY Lv YG Wang L Shi SJ Yang F Zheng GX. Predictive Value of HLA-G and HLA-E in the Prognosis of Colorectal Cancer Patients. Cell Immunol (2015) 293(1):10–6. 10.1016/j.cellimm.2014.10.003 25461612

[33] Kirana C Ruszkiewicz A Stubbs RS Hardingham JE Hewett PJ Maddern GJ. Soluble HLA-G is a Differential Prognostic Marker in Sequential Colorectal Cancer Disease Stages. Int J Cancer (2017) 140(11):2577–86. 10.1002/ijc.30667 28233298

[34] Li JB Ruan YY Hu B Dong SS Bi TN Lin A. Importance of the Plasma Soluble HLA-G Levels for Prognostic Stratification With Traditional Prognosticators in Colorectal Cancer. Oncotarget (2017) 8(30):48854–62. 10.18632/oncotarget.16457 PMC5564730 28415627

[35] Samadi R Nazemalhosseini Mojarad E Molaei M Kazerouni F Asadzadeh Aghdaei H Navidinia M. Clinical Value of Human Leucocyte Antigen G (HLA-G) Expression in the Prognosis of Colorectal Cancer. Int J Cancer Manage (2017) 10(4):e9346. 10.5812/ijcm.9346

[36] Zhang RL Zhang X Dong SS Hu B Han QY Zhang JG. Predictive Value of Different Proportion of Lesion HLA-G Expression in Colorectal Cancer. Oncotarget (2017) 8(64):107441–51. 10.18632/oncotarget.22487 PMC5746078 29296176

[37] Khodabandeh Shahraki P Zare Y Azarpira N Hosseinzadeh M Farjadian S. Prognostic Value of HLA-G in Malignant Liver and Pancreas Lesions. Iran J Immunol (2018) 15(1):28–37. 10.31557/apjcb.2018.3.2.37-45 29549230

[38] Lin A Zhang X Zhang RL Zhang JG Zhou WJ Yan WH. Clinical Significance of Potential Unidentified HLA-G Isoforms Without α1 Domain But Containing Intron 4 in Colorectal Cancer Patients. Front Oncol (2018) 8:361. 10.3389/fonc.2018.00361 30234020 PMC6131604

[39] Murdaca G Calamaro P Lantieri F Pigozzi S Mastracci L Grillo F. HLA-G Expression in Gastric Carcinoma: Clinicopathological Correlations and Prognostic Impact. Virchows Arch (2018) 473(4):425–33. 10.1007/s00428-018-2379-0 29845360

[40] Leelawat K Engprasert S Pongchai-rerk U Tuchinda S Suthipintawong C Leardkamolkarn V. No Expression of Human Leukocyte Antigen G (HLA-G) in Colorectal Cancer Cells. J Med Assoc Thai (2004) 87(7):816–8. 15521238

[41] Ishigami S Natsugoe S Miyazono F Nakajo A Tokuda K Matsumoto M. HLA-G Expression in Gastric Cancer. Anticancer Res (2006) 26(3b):2467–72. 16821634

[42] Wan R Wang ZW Li H Peng XD Liu GY Ou JM. Human Leukocyte Antigen-G Inhibits the Anti-Tumor Effect of Natural Killer Cells Via Immunoglobulin-Like Transcript 2 in Gastric Cancer. Cell Physiol Biochem (2017) 44(5):1828–41. 10.1159/000485819 29224003

[43] Fukushima Y Oshika Y Nakamura M Tokunaga T Hatanaka H Abe Y. Increased Expression of Human Histocompatibility Leukocyte Antigen-G in Colorectal Cancer Cells. Int J Mol Med (1998) 2(3):349–51. 10.3892/ijmm.2.3.349 9855710

[44] Mlecnik B Bindea G Pages F Galon J. Tumor Immunosurveillance in Human Cancers. Cancer Metastasis Rev (2011) 30(1):5–12. 10.1007/s10555-011-9270-7 21249426 PMC3044219

[45] Cai Z Wang L Han Y Gao W Wei X Gong R. Immunoglobulin−Like Transcript 4 and Human Leukocyte Antigen−G Interaction Promotes the Progression of Human Colorectal Cancer. Int J Oncol (2019) 54(6):1943–54. 10.3892/ijo.2019.4761 PMC6521940 30942436

[46] Jiao F Zhou J Sun H Song X Song Y. Plasma Soluble Human Leukocyte Antigen G Predicts the Long-Term Prognosis in Patients With Colorectal Cancer. Trans Cancer Res (2020) 9(6):4011–9. 10.21037/tcr-20-2211 PMC8799004 35117768

[47] Du L Xiao X Wang C Zhang X Zheng N Wang L. Human Leukocyte Antigen-G is Closely Associated With Tumor Immune Escape in Gastric Cancer by Increasing Local Regulatory T Cells. Cancer Sci (2011) 102(7):1272–80. 10.1111/j.1349-7006.2011.01951.x 21466615

[48] Zheng J Xu C Chu D Zhang X Li J Ji G. Human Leukocyte Antigen G is Associated With Esophageal Squamous Cell Carcinoma Progression and Poor Prognosis. Immunol Lett (2014) 161(1):13–9. 10.1016/j.imlet.2014.04.007 24768599

[49] Xu H Shangmian Y Dandan D Ke L. Expression of Human Leukocyte Antígen-G in Esophageal Squamous Cell Carcinoma and its Significance. Cancer Res Clin (2018) 30(9):581–5. 10.3760/cma.j.issn.l006-9801.2018.09.002

[50] Hu J Li L Liu Y Chen Y Liu C Liang W. Overexpression of HLA-G is Positively Associated With Kazakh Esophageal Squamous Cell Carcinoma in Xinjiang, China. Viral Immunol (2013) 26(3):180–4. 10.1089/vim.2012.0085 23772974

[51] Cai MY Xu YF Qiu SJ Ju MJ Gao Q Li YW. Human Leukocyte Antigen-G Protein Expression is an Unfavorable Prognostic Predictor of Hepatocellular Carcinoma Following Curative Resection. Clin Cancer Res (2009) 15(14):4686–93. 10.1158/1078-0432.ccr-09-0463 19584149

[52] Zhou L Niu ZY Liang ZY Zhou WX You L Wang MY. HLA-G Impairs Host Immune Response and Predicts Poor Prognosis in Pancreatic Cancer. Am J Transl Res (2015) 7(10):2036–44. PMC4656780 26692947

[53] Hiraoka N Ino Y Hori S Yamazaki-Itoh R Naito C Shimasaki M. Expression of Classical Human Leukocyte Antigen Class I Antigens, HLA-E and HLA-G, is Adversely Prognostic in Pancreatic Cancer Patients. Cancer Sci (2020) 111(8):3057–70. 10.1111/cas.14514 PMC7419048 32495519

[54] Jiang Y Lu J Wu YE Zhao X Li L. Genetic Variation in the HLA-G 3’UTR 14-Bp Insertion/Deletion and the Associated Cancer Risk: Evidence From 25 Case-Control Studies. Biosci Rep (2019) 39(5):BSR20181991. 10.1042/bsr20181991 30962267 PMC6509057

[55] Akbari A Majd HM Rahnama R Heshmati J Morvaridzadeh M Agah S. Cross-Talk Between Oxidative Stress Signaling and microRNA Regulatory Systems in Carcinogenesis: Focused on Gastrointestinal Cancers. Biomed Pharmacother (2020) 131:110729. 10.1016/j.biopha.2020.110729 33152911

[56] Tan Z Randall G Fan J Camoretti-Mercado B Brockman-Schneider R Pan L. Allele-Specific Targeting of microRNAs to HLA-G and Risk of Asthma. Am J Hum Genet (2007) 81(4):829–34. 10.1086/521200 PMC2227932 17847008

[57] Stairs DB Bayne LJ Rhoades B Vega ME Waldron TJ Kalabis J. Deletion of p120-catenin Results in a Tumor Microenvironment With Inflammation and Cancer That Establishes it as a Tumor Suppressor Gene. Cancer Cell (2011) 19(4):470–83. 10.1016/j.ccr.2011.02.007 PMC3077713 21481789

[58] Farjadian S Tabebordbar M Mokhtari M Safaei A Malekzadeh M Ghaderi A. HLA-G Expression in Tumor Tissues and Soluble HLA-G Plasma Levels in Patients With Gastrointestinal Cancer. Asian Pac J Cancer Prev (2018) 19(10):2731–5. 10.22034/apjcp.2018.19.10.2731 PMC6291033 30360598

[59] Debreova M Csaderova L Burikova M Lukacikova L Kajanova I Sedlakova O. CAIX Regulates Invadopodia Formation Through Both a Ph-Dependent Mechanism and Interplay With Actin Regulatory Proteins. Int J Mol Sci (2019) 20(11):2745. 10.3390/ijms20112745 PMC6600150 31167468

[60] Veo B Danis E Pierce A Sola I Wang D Foreman NK. Combined Functional Genomic and Chemical Screens Identify SETD8 as a Therapeutic Target in MYC-driven Medulloblastoma. JCI Insight (2019) 4(1):e122933. 10.1172/jci.insight.122933 PMC6485357 30626740

