# Supplementary material for: Prognostic and Clinicopathological Value of Human Leukocyte Antigen G in Gastrointestinal Cancers: A Meta-Analysis
Source: Front Oncol. 2021 May 12;11:642902. doi: 10.3389/fonc.2021.642902 (PMC8149900; doi:10.3389/fonc.2021.642902)
Supplement: Supplementary Figure 1 — (A, C) Subgroup analysis of the correlation between HLA-G expression and overall survival (OS) in patients with gastrointestinal (GI) cancer according to the different cancer types. (B, D) Subgroup analysis of the correlation of HLA-G expression with OS in patients with GI cancer according to the different antibodies used for detection. [file DataSheet_1.zip › Table S2.docx]

Table 1 PubMed search strategy (assessed on April 1 2021):

| **Search No.** | **Search terms** | **No. of hits** |
| --- | --- | --- |
| #1 | Search " Colonic Neoplasms"[Mesh] | [76,229] |
| #2 | Search "Rectal Neoplasms"[Mesh] | [48,384] |
| #3 | Search "Colorectal Neoplasms"[Mesh] | [207,593] |
| #4 | Search "Stomach Neoplasms"[Mesh] | [98,832] |
| #5 | Search "Esophageal Neoplasms"[Mesh] | [52,017] |
| #6 | Search "Pancreatic Neoplasms"[Mesh] | [78,582] |
| #7 | Search "Liver Neoplasms"[Mesh] | [170,054] |
| #8 | Search "Gastrointestinal Neoplasms"[Mesh] | [389,881] |
| #9 | Search: (((((((((colon[Title/Abstract]) OR (rectal[Title/Abstract])) OR (rectum[Title/Abstract])) OR (colorectal[Title/Abstract])) OR (stomach[Title/Abstract])) OR (gastric[Title/Abstract])) OR (Esophageal[Title/Abstract])) OR (Pancreatic[Title/Abstract])) OR (Liver[Title/Abstract])) OR (Gastrointestinal[Title/Abstract]) | [1,922,449] |
| #10 | Search: ((((neoplasm*[Title/Abstract]) OR (Neoplasms*[Title/Abstract])) OR (Neoplasms*[Title/Abstract])) OR (carcinoma*[Title/Abstract])) OR (adenocarcinoma*[Title/Abstract]) | [2,128,647] |
| #11 | #9 AND #10 | [673,574] |
| #12 | #1 OR #2 OR #3 OR #4 OR #5 OR #6 OR #7 OR #8 OR #11 | [777,576] |
| #13 | Search "HLA-G Antigens"[Mesh] | [1,865] |
| #14 | Search ("HLA-G"[Title/Abstract] OR "HLA G"[Title/Abstract] OR "human leukocyte antigen G"[Title/Abstract]) | [2,409] |
| #15 | #13 OR #14 | [2,554] |
| #16 | #12 AND #15 | [97] |

**Search strategy: ((((((((("Colonic Neoplasms"[Mesh]) OR ("Rectal Neoplasms"[Mesh])) OR ("Colorectal Neoplasms"[Mesh])) OR ("Stomach Neoplasms"[Mesh])) OR ("Esophageal Neoplasms"[Mesh])) OR ("Pancreatic Neoplasms"[Mesh])) OR ("Liver Neoplasms"[Mesh])) OR ("Gastrointestinal Neoplasms"[Mesh])) OR (((((((((((colon[Title/Abstract]) OR (rectal[Title/Abstract])) OR (rectum[Title/Abstract])) OR (colorectal[Title/Abstract])) OR (stomach[Title/Abstract])) OR (gastric[Title/Abstract])) OR (Esophageal[Title/Abstract])) OR (Pancreatic[Title/Abstract])) OR (Liver[Title/Abstract])) OR (Gastrointestinal[Title/Abstract])) AND (((((neoplasm*[Title/Abstract]) OR (Neoplasms*[Title/Abstract])) OR (tumor*[Title/Abstract])) OR (carcinoma*[Title/Abstract])) OR (adenocarcinoma*[Title/Abstract])))) AND (("HLA-G Antigens"[Mesh]) OR (((HLA-G[Title/Abstract]) OR (HLA G[Title/Abstract])) OR (human leukocyte antigen G[Title/Abstract])))**

Table 2 Web of science search strategy (assessed on April 1 2021):

| **Search No.** | **Search terms** | **No. of hits** |
| --- | --- | --- |
| #1 | Search: ((((((((((colon*[Title/Abstract]) OR (rectal[TOPIC])) OR (rectum[TOPIC])) OR (colorectal[TOPIC])) OR (stomach[TOPIC])) OR (gastric[TOPIC])) OR (Esophageal[TOPIC])) OR (Pancreatic[TOPIC])) OR (Liver[TOPIC])) OR (Gastrointestinal[TOPIC]) | [404,723] |
| #2 | Search (neoplasm*[TOPIC] OR Neoplasms*[TOPIC] OR tumor*[TOPIC] OR carcinoma*[TOPIC] OR adenocarcinoma*[TOPIC]) | [587,103] |
| #3 | #1 OR #2 | [967,376] |
| #4 | Search ("HLA-G"[TOPIC] OR "HLA G"[TOPIC] OR "human leukocyte antigen G"[TOPIC]) | [9,761] |
| #5 | #3 AND #4 | [386] |

Table 3 Embase search strategy (assessed on April 1 2021):

| **Search No.** | **Search terms** | **No. of hits** |
| --- | --- | --- |
| #1 | Search 'colon tumor'/exp | [350,644] |
| #2 | Search 'rectum tumor'/exp | [279,921] |
| #3 | Search 'colorectal tumor'/exp | [32,179] |
| #4 | Search 'stomach tumor'/exp | [170,680] |
| #5 | Search 'esophagus tumor'/exp | [93,025] |
| #6 | Search 'pancreas tumor'/exp | [162,865] |
| #7 | Search 'liver tumor'/exp | [310,887] |
| #8 | Search 'gastrointestinal tumor'/exp | [29,800] |
| #9 | Search colon:ab,ti OR rectal:ab,ti OR rectum:ab,ti OR colorectal:ab,ti OR stomach:ab,ti OR esophageal:ab,ti OR pancreatic:ab,ti OR liver:ab,ti OR gastrointestinal:ab,ti | [2,385,971] |
| #10 | Search 'neoplasm*':ab,ti OR 'cancer*':ab,ti OR 'Neoplasms*':ab,ti OR 'carcinoma*':ab,ti OR 'adenocarcinoma*':ab,ti | [3,369,795] |
| #11 | #9AND #10 | [762,314] |
| #12 | #1 OR #2 OR #3 OR #4 OR #5 OR #6 OR #7 OR #8 OR #11 | [1,210,102] |
| #13 | Search 'hla g antigen'/exp | [3,025] |
| #14 | Search 'human leukocyte antigen g':ab,ti OR 'human leukocyte antigen-g':ab,ti OR 'hla-g':ab,ti OR 'hla g':ab,ti OR 'human leukocyte antigen g'/exp OR 'human leukocyte antigen g' OR 'human leukocyte antigen-g' | [3,525] |
| #15 | #13 OR #14 | [4,207] |
| #16 | #12 AND #15 | [181] |

Table 4 Cochrane search strategy (assessed on April 1 2021):

| **Search No.** | **Search terms** | **No. of hits** |
| --- | --- | --- |
| #1 | Search "Colonic Neoplasms"[Mesh] | [1,763] |
| #2 | Search "Rectal Neoplasms"[Mesh] | [1,861] |
| #3 | Search "Colorectal Neoplasms"[Mesh] | [8,413] |
| #4 | Search "Stomach Neoplasms"[Mesh] | [2,637] |
| #5 | Search "Esophageal Neoplasms"[Mesh] | [1,637] |
| #6 | Search "Pancreatic Neoplasms"[Mesh] | [1,795] |
| #7 | Search "Liver Neoplasms"[Mesh] | [3,007] |
| #8 | Search "Gastrointestinal Neoplasms"[Mesh] | [12,989] |
| #9 | Search: (((((((((colon[Title/Abstract]) OR (rectal[Title/Abstract])) OR (rectum[Title/Abstract])) OR (colorectal[Title/Abstract])) OR (stomach[Title/Abstract])) OR (gastric[Title/Abstract])) OR (Esophageal[Title/Abstract])) OR (Pancreatic[Title/Abstract])) OR (Liver[Title/Abstract])) OR (Gastrointestinal[Title/Abstract]) | [169,038] |
| #10 | Search: ((((neoplasm* [Title/Abstract]) OR (Neoplasms*[Title/Abstract])) OR (Neoplasms* [Title/Abstract])) OR (carcinoma*[Title/Abstract])) OR (adenocarcinoma*[Title/Abstract]) | [108,026] |
| #11 | #9 AND #10 | [35,114] |
| #12 | #1 OR #2 OR #3 OR #4 OR #5 OR #6 OR #7 OR #8 OR #11 | [35,752] |
| #13 | Search "HLA-G Antigens"[Mesh] | [6] |
| #14 | Search ("HLA-G"[Title/Abstract] OR "HLA G"[Title/Abstract] OR "human leukocyte antigen G"[Title/Abstract]) | [982] |
| #15 | #13 OR #14 | [982] |
| #16 | #12 AND #15 | [31] |
