# Supplementary material for: Prognostic and Clinicopathological Value of Human Leukocyte Antigen G in Gastrointestinal Cancers: A Meta-Analysis
Source: Front Oncol. 2021 May 12;11:642902. doi: 10.3389/fonc.2021.642902 (PMC8149900; doi:10.3389/fonc.2021.642902)
Supplement: Supplementary Figure 1 — (A, C) Subgroup analysis of the correlation between HLA-G expression and overall survival (OS) in patients with gastrointestinal (GI) cancer according to the different cancer types. (B, D) Subgroup analysis of the correlation of HLA-G expression with OS in patients with GI cancer according to the different antibodies used for detection. [file DataSheet_1.zip › table S3.docx]

Table S2 Quantitative Assessment for Publication Bias

| Quantitative Assessment for Publication Bias | | | | | | | | | |
| --- | --- | --- | --- | --- | --- | --- | --- | --- | --- |
|  | Pos VS Neg  (UNI) | Pos VS Neg  (MUL) | Strong VS Weak  (UNI) | Strong VS Weak  (MUL) | Clinical  Stage | T | N | M | histological grade |
| Number of studies | 13 | 12 | 4 | 6 | 13 | 20 | 23 | 9 | 25 |
| P value for  Begg's Test | 0.009 | 0.020 | 1.000 | 0.707 | 0.009 | 0.105 | 0.010 | 0.048 | 0.815 |
| P value for  Egger's test | 0.000 | 0.002 | 0.796 | 0.819 | 0.000 | 0.455 | 0.059 | 0.000 | 0.344 |

**Abbreviations:** T, tumor status; N, nodal status; M, metastasis; Uni, univariate; MUL, multivariate.
