# Supplementary figures and images for: Prognostic and Clinicopathological Value of Human Leukocyte Antigen G in Gastrointestinal Cancers: A Meta-Analysis
Source: Front Oncol. 2021 May 12;11:642902. doi: 10.3389/fonc.2021.642902 (PMC8149900; doi:10.3389/fonc.2021.642902)

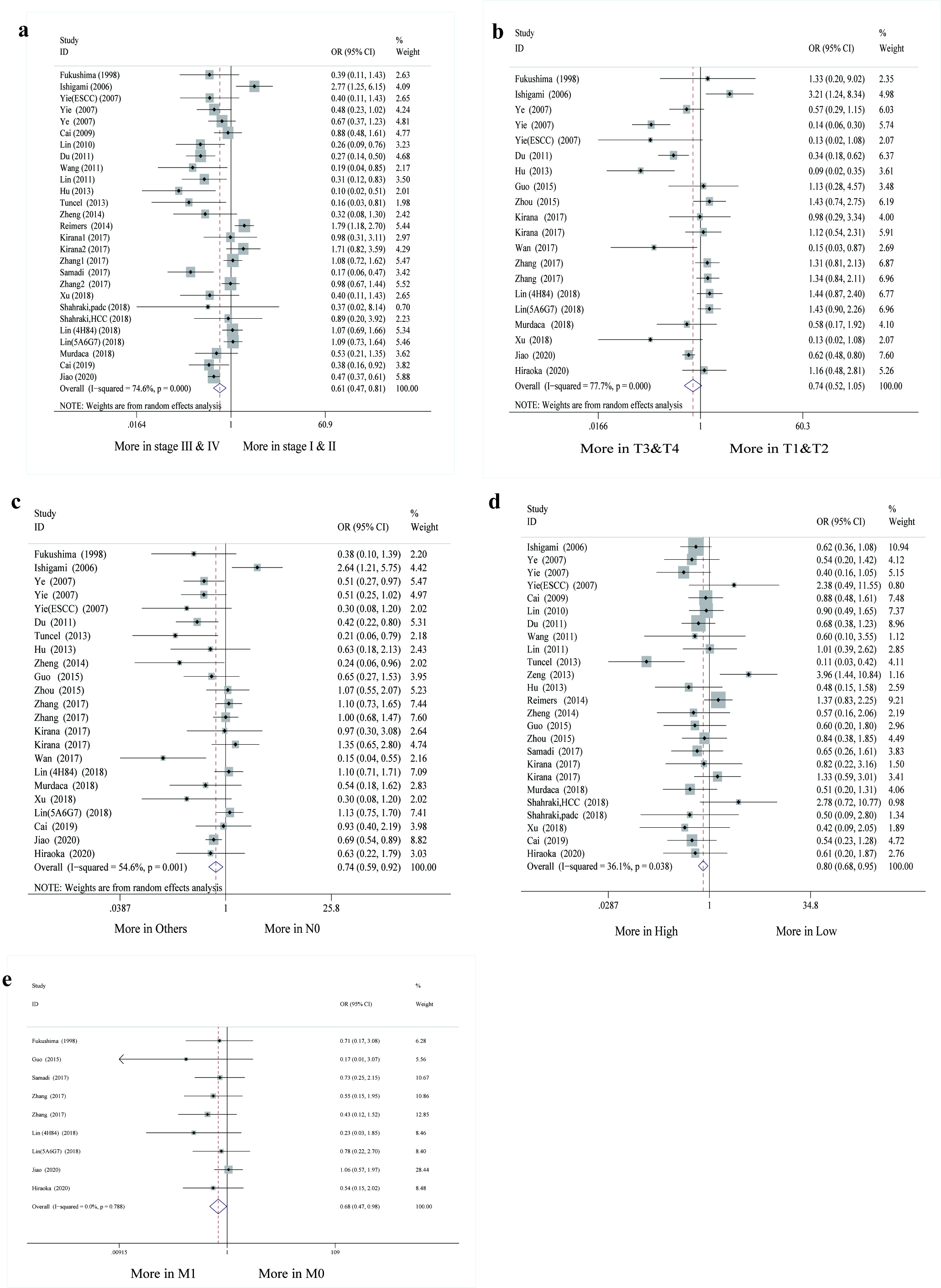

Supplement: Supplementary Figure 1 — (A, C) Subgroup analysis of the correlation between HLA-G expression and overall survival (OS) in patients with gastrointestinal (GI) cancer according to the different cancer types. (B, D) Subgroup analysis of the correlation of HLA-G expression with OS in patients with GI cancer according to the different antibodies used for detection. [file DataSheet_1.zip › figure s1.TIF]

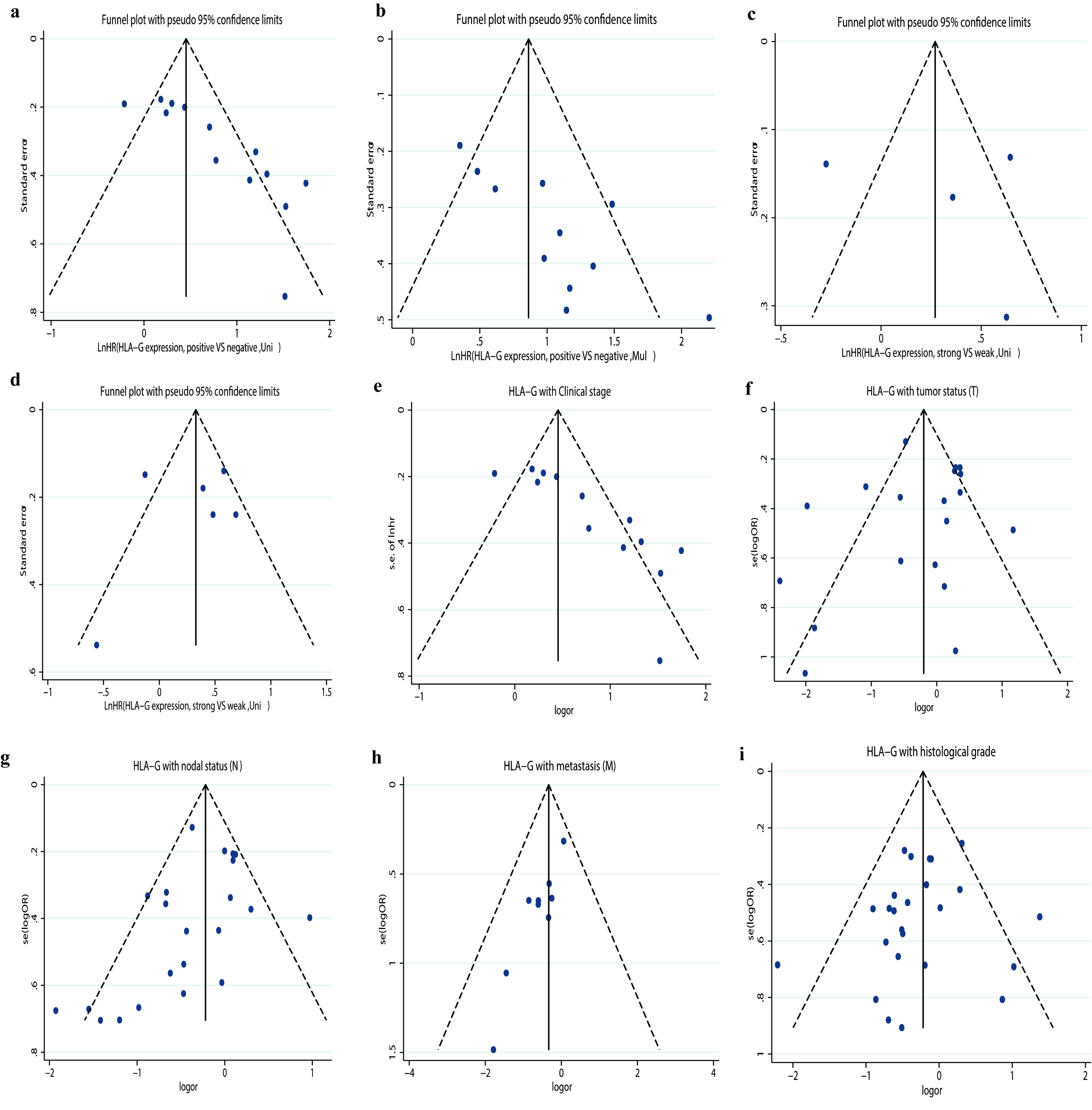

Supplement: Supplementary Figure 1 — (A, C) Subgroup analysis of the correlation between HLA-G expression and overall survival (OS) in patients with gastrointestinal (GI) cancer according to the different cancer types. (B, D) Subgroup analysis of the correlation of HLA-G expression with OS in patients with GI cancer according to the different antibodies used for detection. [file DataSheet_1.zip › figure s3.tif]

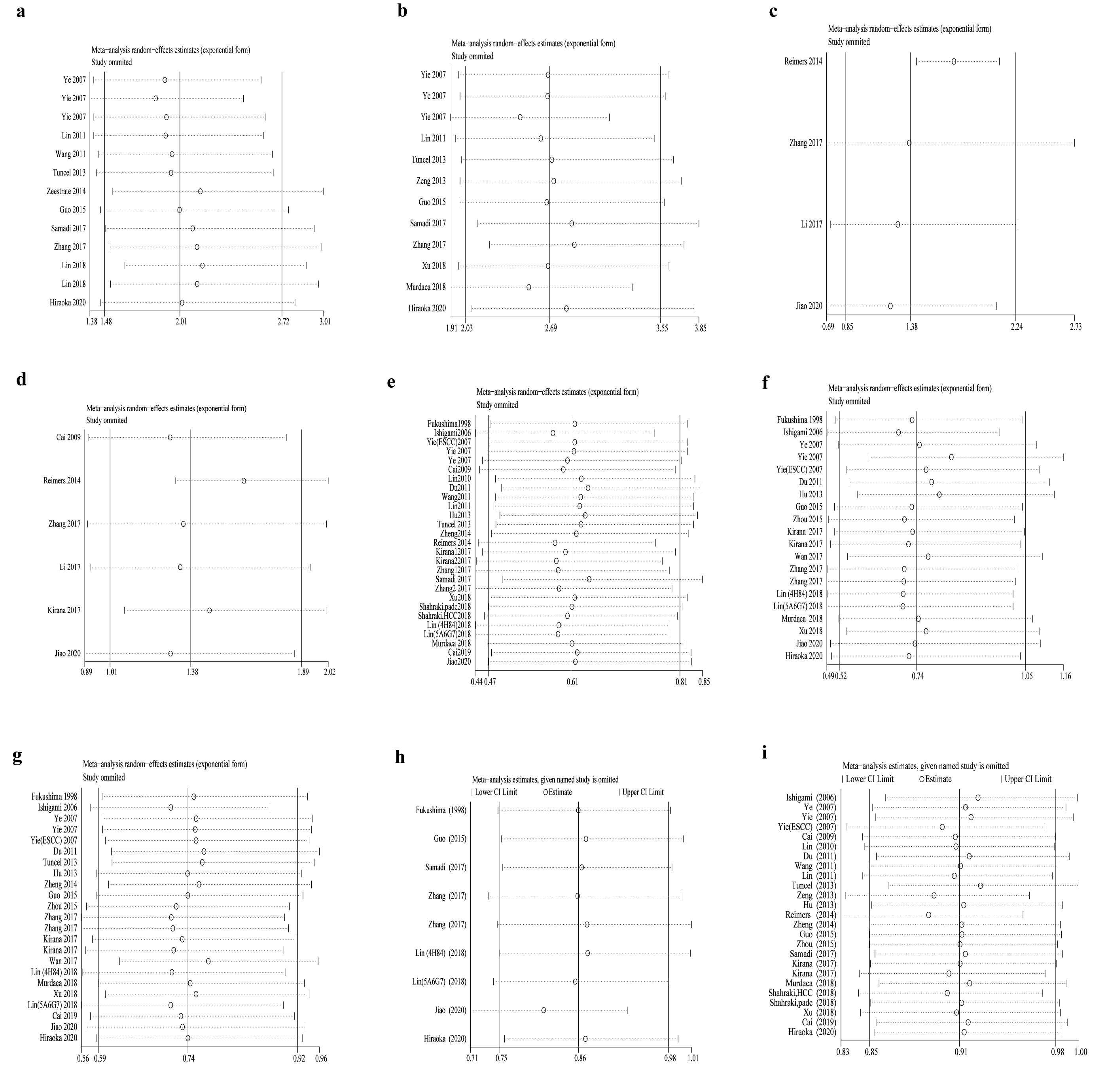

Supplement: Supplementary Figure 1 — (A, C) Subgroup analysis of the correlation between HLA-G expression and overall survival (OS) in patients with gastrointestinal (GI) cancer according to the different cancer types. (B, D) Subgroup analysis of the correlation of HLA-G expression with OS in patients with GI cancer according to the different antibodies used for detection. [file DataSheet_1.zip › figure s4.tif]

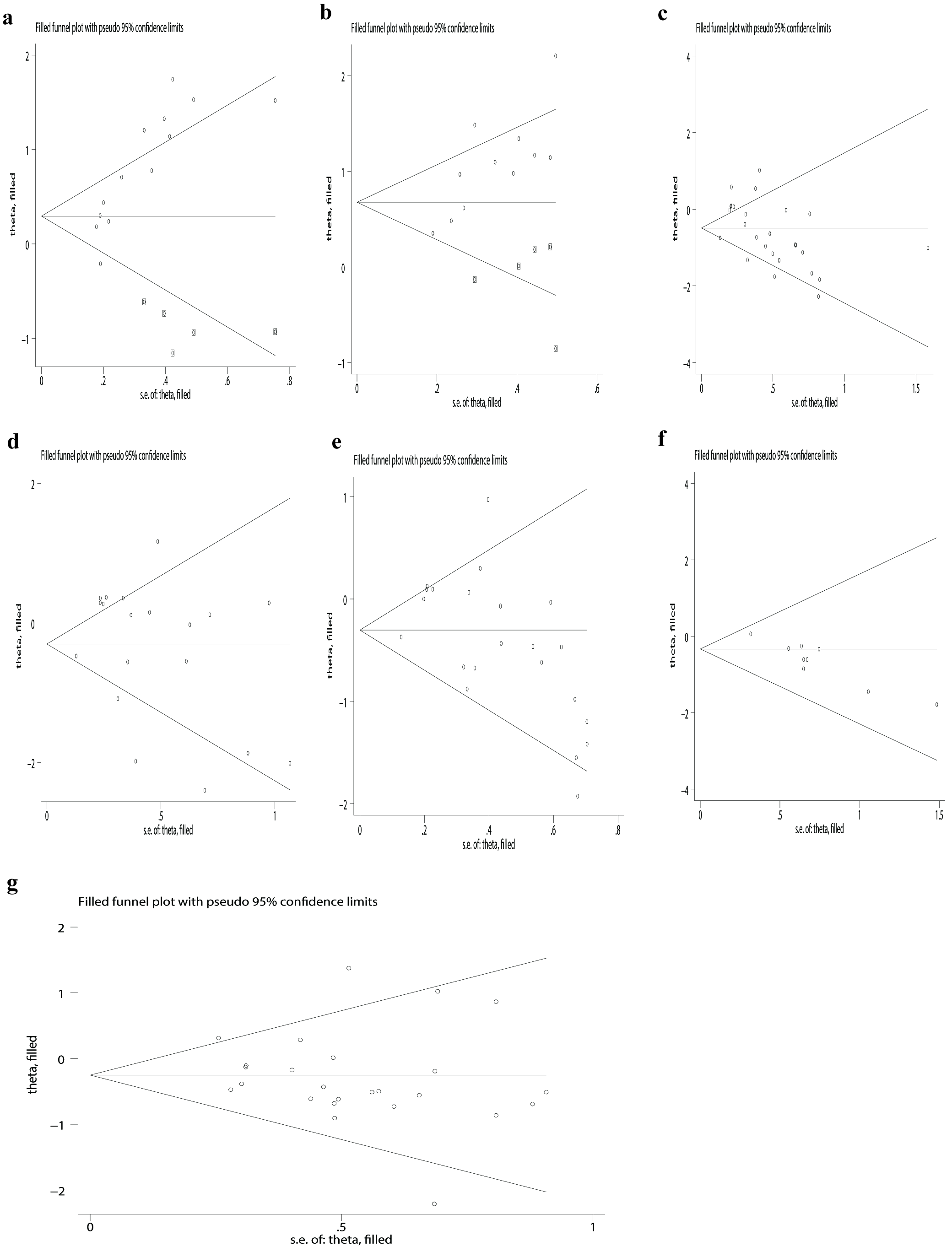

Supplement: Supplementary Figure 1 — (A, C) Subgroup analysis of the correlation between HLA-G expression and overall survival (OS) in patients with gastrointestinal (GI) cancer according to the different cancer types. (B, D) Subgroup analysis of the correlation of HLA-G expression with OS in patients with GI cancer according to the different antibodies used for detection. [file DataSheet_1.zip › figure s5.tif]

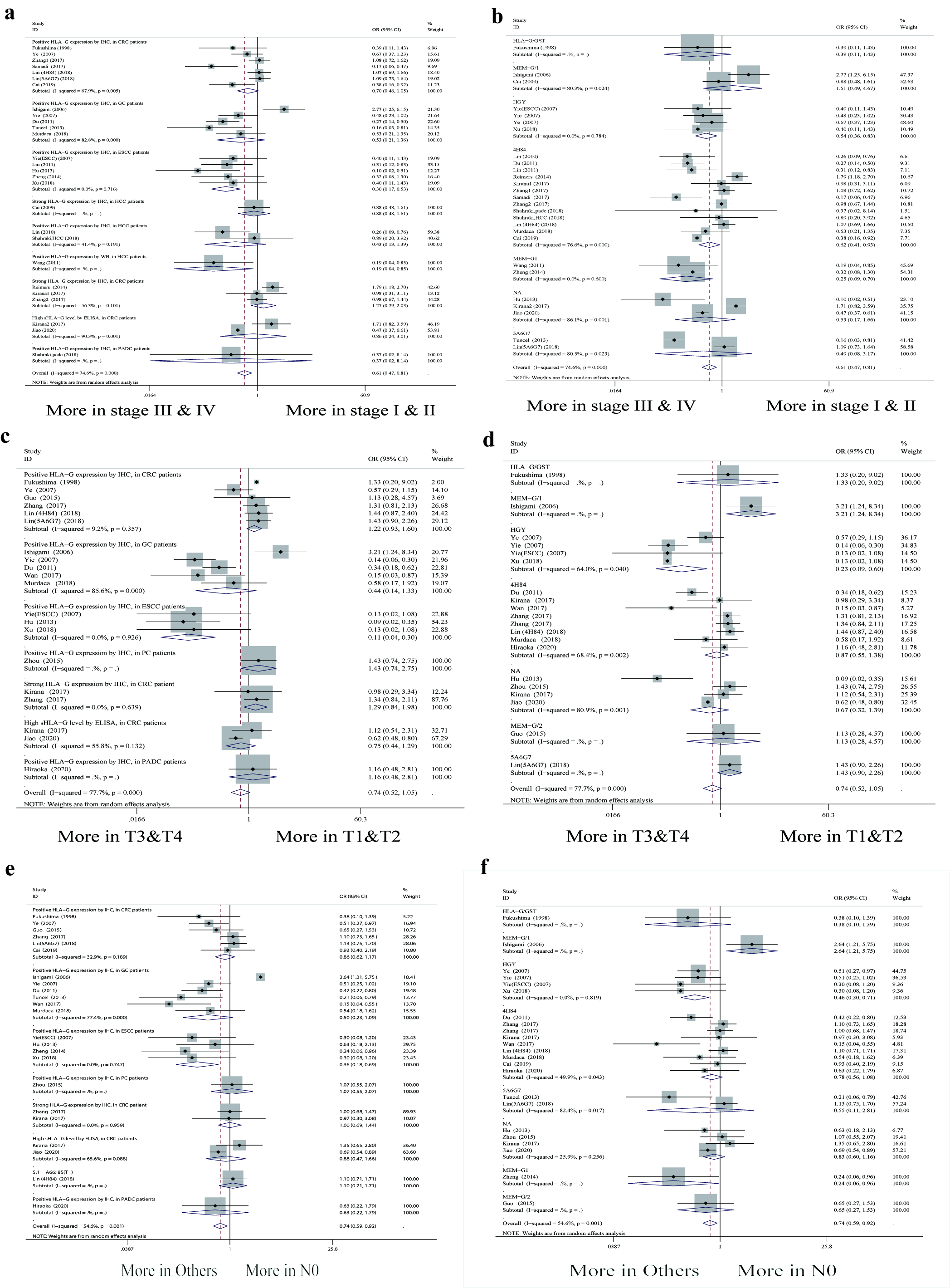

Supplement: Supplementary Figure 1 — (A, C) Subgroup analysis of the correlation between HLA-G expression and overall survival (OS) in patients with gastrointestinal (GI) cancer according to the different cancer types. (B, D) Subgroup analysis of the correlation of HLA-G expression with OS in patients with GI cancer according to the different antibodies used for detection. [file DataSheet_1.zip › FIGURES2.tif]
